# High SGO2 predicted poor prognosis and high therapeutic value of lung adenocarcinoma and promoted cell proliferation, migration, invasion, and epithelial-to-mesenchymal transformation

**DOI:** 10.7150/jca.86285

**Published:** 2023-07-24

**Authors:** Zuotao Wu, Ting Zhuo, Zihao Li, Yongjie Zhu, Jiejing Wu, Guanbiao Liang, Lei Dai, Yongyong Wang, Xiang Tan, Mingwu Chen

**Affiliations:** 1 Department of Cardio-Thoracic Surgery, The First Affiliated Hospital of Guangxi Medical University, Nanning, Guangxi, China.; 2 Department of Pulmonary and Critical Care Medicine, The First Affiliated Hospital of Guangxi Medical University, Nanning, Guangxi, China.; 3 Department of Ophthalmology, Affiliated Hospital of Youjiang Medical University for Nationalities, Baise, Guangxi, China.

**Keywords:** SGO2, LUAD, prognostic markers, cell proliferation, drug sensitivity

## Abstract

**Background:** Shugoshin 2 (SGO2), a component of the cell division cohesion complex, is involved in both mitotic and meiotic processes. Despite being overexpressed in various malignant tumors and is associated with poor prognosis, its exact role in lung adenocarcinoma (LUAD) and its biological effects on lung cancer cells are not well understood.

**Methods:** The transcriptomics data and clinical information for LUAD were obtained from TCGA and GEO, and DEGs associated with prognostic risk factors were screened using Cox regression analysis and chi-square testing. Identify these gene functions using correlation heatmaps, protein interaction networks (PPIs), and KEGG enrichment assays. The expression of SGO2 in tissues was verified by PCR and IHC, and the prognostic value of SGO2 in LUAD was evaluated by survival analysis. In addition, the effects of SGO2 knockdown on lung cancer cell proliferation, migration, invasion, and epithelial-to-mesenchymal transition (EMT) were studied in vitro. After that, the TIMER database and single-sample GSEA (ssGSEA) analysis were used to investigate the correlation between SGO2 and immune infiltration. Finally, the tumor mutational burden (TMB) of different SGO2 clusters and the efficacy of the two clusters in multiple treatments were evaluated.

**Results:** High-risk genes associated with poor prognosis in LUAD are involved in cell cycle regulation and proliferation. Among these genes, SGO2 exhibited high expression in LUAD and corresponded with the TNM stage. Furthermore, the knockdown of SGO2 led to a decrease in the proliferation, migration, invasion, and EMT processes of lung cancer cells. Notably, high SGO2 expression may have poorer anti-tumor immunity and may therefore be more suitable for immunotherapy to re-establish immune function, while its high expression with a higher TMB could enable LUAD to benefit from multiple therapies.

**Conclusion:** Our findings suggest that SGO2 may be a promising prognostic biomarker for LUAD, particularly in regulating the cell cycle and benefiting from multiple therapies.

## Introduction

Non-small cell lung cancer is a major global health challenge and a serious threat to human life, particularly lung adenocarcinoma (LUAD), one of the most malignant tumors arising from epithelial-derived stem cell mutations.^1^, ^2^ Despite early radical resection, LUAD remains a heterogeneous tumor type with a high risk of recurrence.^3^ The mechanisms underlying early metastasis and immune evasion in the disease are not well understood. Therefore, we utilized bioinformatics analysis of LUAD RNA-Seq transcript and clinical data from The Cancer Genome Atlas (TCGA) to investigate the relationship between patient prognosis and clinical grading. Our findings revealed significant upregulation of SGO2, a centromere-associating protein that protects against premature cleavage of sister chromatids during cell division. Given that tumor cells proliferate rapidly and grow, SGO2 may play an important role in delaying chromosomal separation to promote early metastasis and immune evasion.

The Shugoshin family consists of two evolutionarily conserved proteins, Shugoshin 1 (SGO1) and Shugoshin 2 (SGO2). SGO1 and SGO2 mediate homodimerization through the N-terminal helical coil structure and localize to the centromere using the C-terminal base.^4^ Interestingly, SGO2 not only maintains centromere cohesion during mitosis but also assists in precise localization of the chromosomes passenger complex (CPC) to the centromere and subsequent catalysis of the Aurora B kinase, thereby ensuring appropriate chromosomal alignment.^5^ Moreover, SGO2 is enriched at subtelomeres in the G2 phase, which promotes the expression of heat shock protein 70 (HSP70) alongside heat shock transcription factor 1 (HSF1) in case of cellular injury, thereby sustaining cell survival.^6^, ^7^ As a result of its interactions with various proteins that mediate cell division, chromosome segregation, and the cell cycle, SGO2 co-regulates chromosome separation and alters cell proliferation and cycle stability.

To further investigate the potential of SGO2 as a prognostic marker for LUAD, we evaluated its expression and distribution in tumor samples via RT-qPCR and IHC. Additionally, we utilized small interfering RNA (siRNA) to knock out SGO2 in lung cancer cell lines, confirming its effect on proliferation, migration, invasion, and epithelial-to-mesenchymal transition (EMT). To verify the relationship between SGO2 and tumor-infiltrating immune cells, it may become a new therapeutic target for LUAD. Finally, the effect of the high expression of SGO2 on therapeutic efficacy was also verified.

## Materials & Methods

### Data mining and screening for high-risk genes

The RNA-Seq transcriptome and clinical data were gathered from TCGA. Proportional risk regression analysis was used to examine the association between overall survival (OS) and age, sex, and TNM stage, and the relationship between T, N, and stage and OS was demonstrated by the Kaplan-Meier survival curve (K-M curve). The DESeq2 package was used to analyze differentially expressed genes (DEGs) in LUAD, with DEGs expression divided into high and low variables based on the median, and prognostic genes identified through univariate Cox analysis.^8^ The chi-square test was utilized to identify prognostic genes with T, N, and stage. These genes were then screened through logistics regression analysis, and multivariate COX regression was used to construct prognostic models for the selected genes and clinical characteristics to obtain and evaluate the prognostic value of high-risk genes. 9, 10

### PPI, correlation heatmap, and functional enrichment analysis

The interconnections of high-risk genes underwent protein-protein interaction (PPI) analysis, and the PPI network through the online web service, STRING. Node genes were screened from the PPI network and analyzed using KEGG for functional enrichment in DAVID Bioinformatics Resource, and analyze the correlation of node genes using a correlation heatmap.^11^ Venn diagram methods are used to identify genes critical to key pathways. The correlation heatmap is implemented using R, Sankey diagram and Venn diagram and was plotted by https://www.bioinformatics.com.cn (last accessed on 15 Jun 2023), an online platform for data analysis and visualization.

### SGO2 expression and prognosis analysis

SGO2 expression in normal and cancer tissues was examined using the LUAD dataset in TCGA as well as validation datasets GSE30219 and GSE31210. SGO2 was evaluated as a diagnostic and prognostic biomarker for LUAD by constructing ROC curves and K-M curves. Use R to visualize the results.^12^

### LUAD samples and histopathological sections were obtained

From July to September 2022, fresh tumors and adjacent normal tissues were collected from thirty patients diagnosed with invasive LUAD at the Department of Cardio-Thoracic Surgery, The First Affiliated Hospital of Guangxi Medical University. Furthermore, para-cancer and carcinoma histopathological sections from eight LUAD patients from November to December 2022 were obtained from the Department of Pathology, the First Affiliated Hospital of Guangxi Medical University.

### Cell culture and RNA interference

We received human NSCLC cell lines (A549 and H1299) from the Chinese Academy of Sciences (Shanghai, China). A549 and H1299 cells were cultivated in Dulbecco's modified eagle medium (Gibco, Grand Island, USA) supplemented with 10% fetal bovine serum (Gibco), penicillin, and streptomycin. These cell lines were maintained at 37 °C and 5% CO2 and then exposed to siRNA (Nanning Gensis Biotechnology Ltd, China) using Lipofectamine 8000 (Beyotime, China) in a serum-free medium after cells were cultured for 24 hours. The siSGO2 sequence used was 5′-GAACACAUUCUUCGCCUATT-3′, while non-targeted siRNAs were used with the sequence 5′-UUCUCCGAACGUGUCACGU-3′.^13^

### RNA isolation and reverse transcription

Fresh tissue samples or cells were added RNAiso Plus (Takara, Kyoto, Japan) to extract total RNA at 4°C. To generate cDNA, 1.0µg RNA was reverse transcribed using a Prime Script RT Master Mix (Takara) according to the manufacturer's protocol.

### Cell lysate preparation

To extract proteins from the cytoplasm, RIPA Lysis buffer (Beyotime) containing 1% PMSF (Beyotime) was used to lyse the cells. After assessing the protein concentration, add buffer and boil for 10 min before storing at -80 °C.

### Real-Time Quantitative PCR

RT-qPCR was conducted using a 2X Q3 SYBR qPCR Master mix (ToloBio, China) in the Roche LightCycler480II Real-time PCR System to measure gene expression levels. The relative quantitative gene expression of SGO2 and control genes was analyzed using the 2-ΔΔCt method.^14^ SGO2 forward, 5′- ATGTGGTGCATGGCCTAAAAA-3′ and reverse, 5′- GGGGTACATATTGGTGATCTGC-3′; GAPDH forward, 5′-GCACCGTCAAGGCTGAGAAC-3′ and reverse, 5′-ATGGTGGTGAAGACGCCAGT-3′.^15^

### Western blot

25 μg per lane is loaded onto 8% SDS-PAGE for electrophoresis and transferred to a PVDF membrane (Sigma Aldrich, USA). Incubate with the primary antibody overnight at 4 °C. The following primary antibodies were used: E-Cadherin (Cat. # 20874-1-AP; 1:20000; Proteintech, Wuhan, China), N-Cadherin (Cat. # 22018-1-AP; 1:2000; Proteintech), Vimentin (Cat. # abs171412; 1:1000; absin, Shanghai, China), Pan-Cytokeratin (Cat. # BH0149; 1:1000; Bioss, Beijing, China), and SGO2 (Cat. # A30763-1; 1:1000; Boster, Wuhan, China). Normalization was performed with β-Actin (Cat. # 81115-1-RR; 1:5000; Proteintech).

### Immunohistochemical

The paraffin sections were baked, dewaxed, hydrated, and washed. Heat-mediated antigen retrieval was performed using sodium citrate buffer for 20 minutes. Using 3% H2O2 treats peroxidase activity. The sections were incubated with a 1:1000 rabbit anti-human SGO2 antibody (Cat. # NBP1-83567; Novus, USA) overnight at a 4 °C in wet box. Finally, complete the staining with DAB and hematoxylin and mount the coverslip.^16^ We measured the average optical density (AOD) to assess the expression of SGO2 in carcinoma and para-carcinoma, which was the ratio of optical density (OD) and the observed area by using ImageJ.^17^

### Cell proliferation

We performed cell proliferation assays using the Cell counting kit-8 (CCK-8; Beyotime) and BeyoClickTM EdU-555 Cell Proliferation Detection Kit (EdU; Beyotime). Briefly, absorbance was measured at 450 nm by adding CCK-8 solution to the cells at 24, 48, and 72 h. EdU solution was added 24 h after transfection, and cells were observed and photographed using a fluorescence microscope (EVOS M7000, Thermo Fisher Scientific, USA).

### Wound healing assay

Cells are seeded into six-well plates and when the density reaches approximately 80-90%, a vertical wound is created using a sterile 10μL tip. Wash twice and images of the same area were captured using a microscope (Nikon Japan) at 0 h and 24 h.

### Transwell assay

Migration and invasion were conducted using transwell chambers (LABSELECT, China) with 8μm pore size. Cells harvested 24 hours after transfection were resuspended and added to the upper chamber at a volume of 250μL, 700μL of medium containing 10% FBS in the lower chamber, and placed in the incubator for 36 h. After crystal violet (Beyotime) staining, cells are counted using a microscope (Nikon Japan) in several randomly selected regions.

### Tumor immune cell infiltration and ssGSEA

The deconvolution algorithm (CIBERSORT ABS.MODE) was to determine the abundance of 9 types of tumor-infiltrating lymphocytes (TILs) of LUAD in TIMER.^18^ Additionally, ssGSEA analysis was performed on 28 different types of tumor-infiltrating immune cells (TIICs) using the GSVA package.^19^

### Analysis of tumor mutation burden (TMB) and therapeutic effect between the different SGO2 clusters

The R package, maftools, was used to plot oncoplots for the different SGO2 clusters (cutoff value was 50%). Subsequently, the TMB and immune checkpoint expressions of different SGO2 clusters in LUAD were compared.^20^ Another R package, oncoPredict, was used to compare the chemotherapy and targeted therapy efficacy of different SGO2 clusters.^21^

### Visualization and statistical analysis

Based on the changes observed in the image, ImageJ is used for mathematical calculations. Data were analyzed and presented as mean ± standard deviation (SD) using GraphPad Prism 5.0 (GraphPad Software, USA) and SPSS23.0 (IBM, USA). The t-test was used to compare two independent samples, while a single-tailed paired t-test was used to compare carcinoma and para-carcinoma. The Wilcoxon rank sum test (unpaired samples of two groups) were applied to define the differences in clinical information of LUAD parents. Statistical significance was defined as p < 0.05 (ns, p > 0.05; *, p < 0.05; **, p < 0.01; ***, p < 0.001). Each experiment was repeated more than three times.

## Results

### Prognostic factors and prognosis-related genes for LUAD

**Figure 1:**
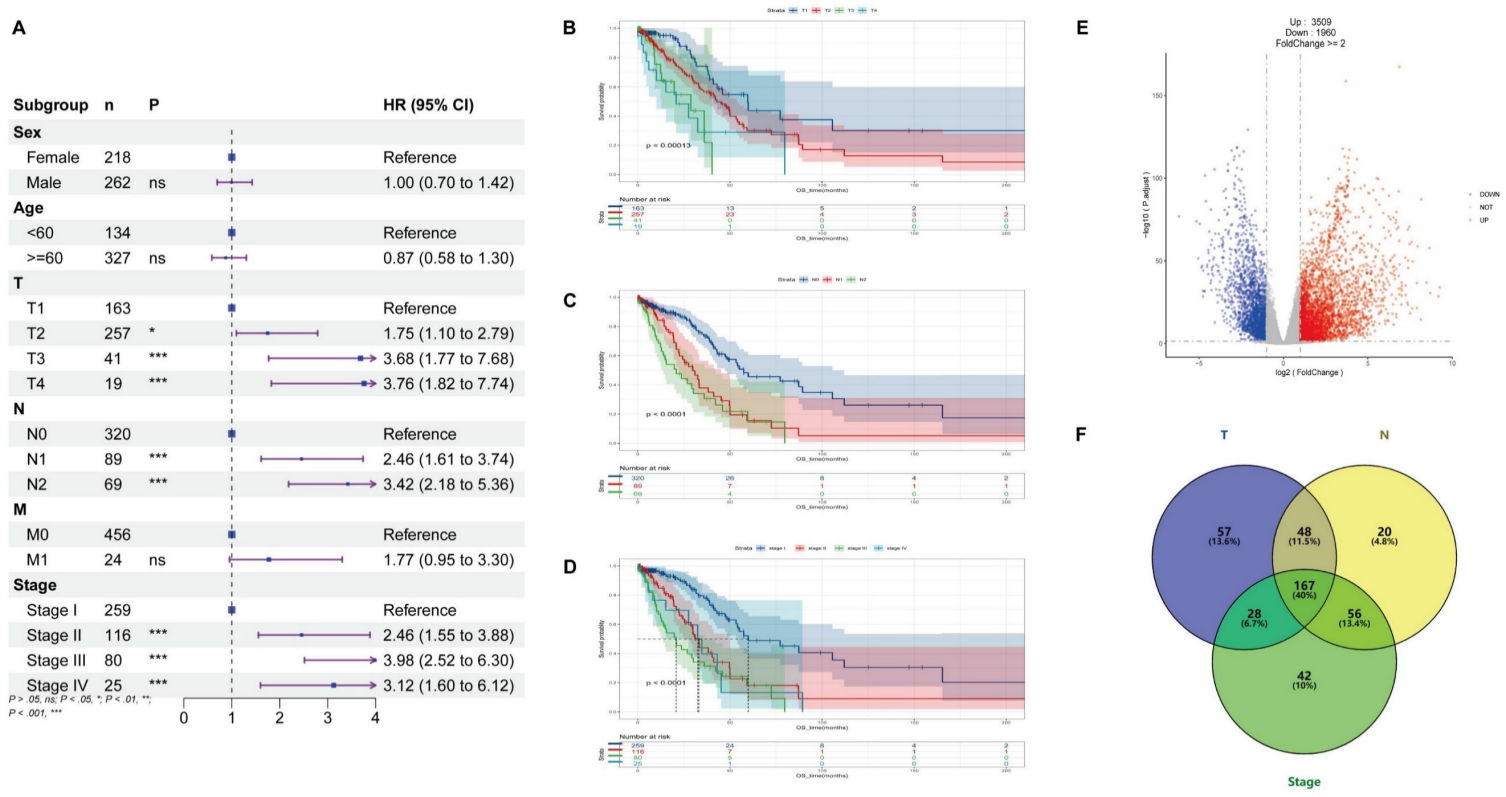
** Prognostic factors and prognosis-related genes for LUAD.** (A) Univariate Cox analysis was conducted using TCGA, T, N, and stage being used as prognostic factors for LUAD. (B-D) According to survival curves for T, N, and stage of LUAD, T1, N0, and Stage-I had longer overall survival (OS) compared to other stages. (E) Differential expression analysis was performed using RNAseq transcriptome of 480 lung adenocarcinoma (LUAD) and 50 normal lung tissues in the TCGA database, and the screening criteria were Foldchange>=2 and P < 0.05, 3,509 expression upregulated genes and 1,960 expression downregulated genes were obtained in LUAD. (F) Based on the chi-square test, we identified 300 T-related prognostic genes, 291 N-related prognostic genes, and 293 Stage-related prognostic genes. The intersection of these genes using the Venn diagram yielded 167 prognostically relevant risk genes.

Clinical information for patients is shown in **Table 1**, and through univariate Cox analysis of age, sex, and TNM stage in the data, it was observed that high T, N and stage were significantly correlated with poor prognosis and demonstrated their effect on overall survival (**Figure 1A-D**). After differential analysis and survival analysis (Foldchange>=2 and P < 0.05, **Figure 1E**), 648 prognostic genes were obtained from 3509 upregulated genes, and based on systematic data mining (HR < 1 & p < 0.05, **Table S1A**), 167 prognostic genes associated with T, N and stage were screened (p < 0.05, **Figure 1F** and **Table S1B-D**), and these prognosis-related genes were further screened until 30 high-risk genes (p < 0.05, **Table S1E-F**).

### The correlation and functional enrichment analysis of high-risk genes

**Figure 2:**
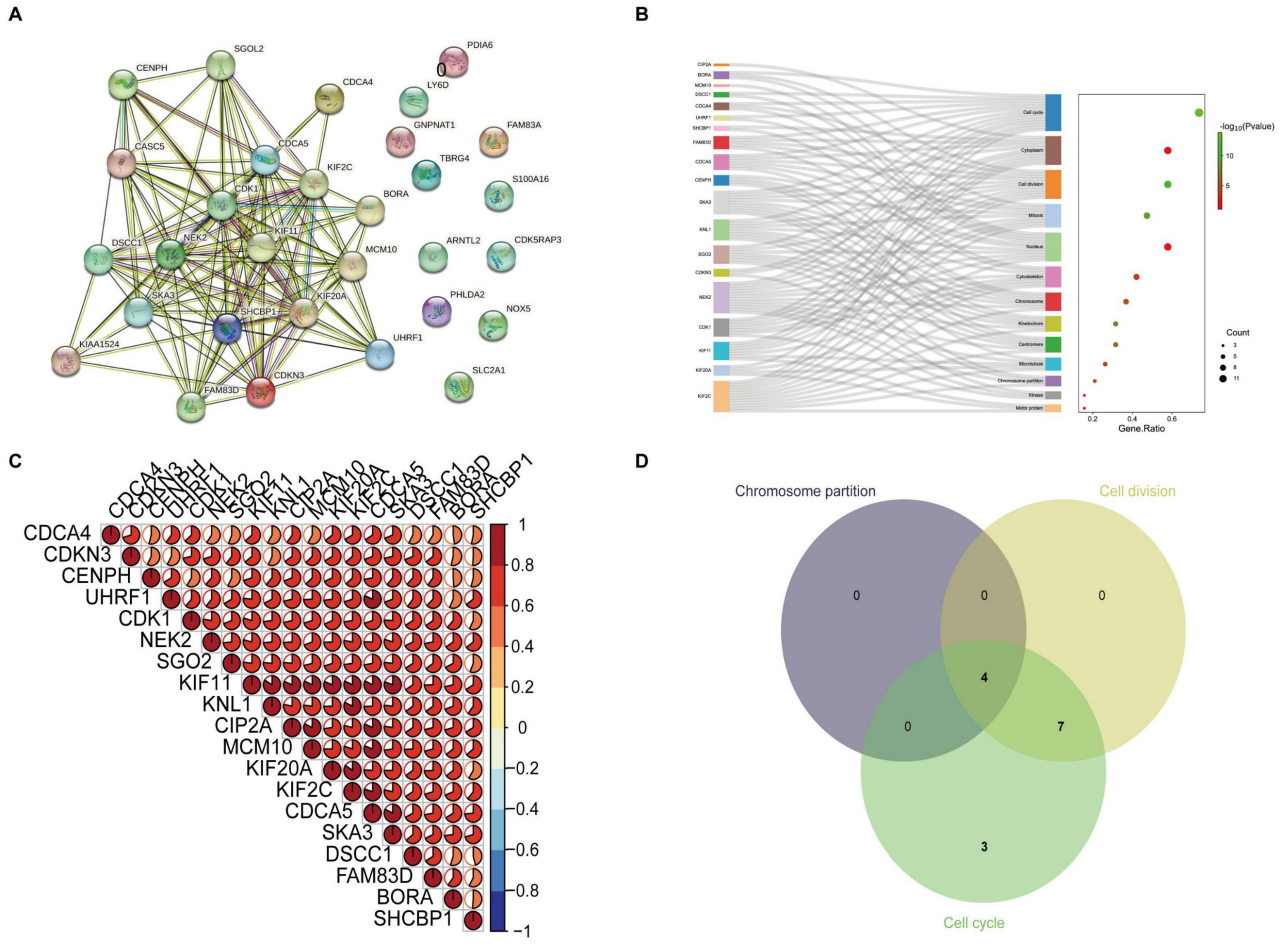
** The correlation and functional enrichment analysis.** (A) Protein-protein interaction networks analysis was performed on nineteen high-risk genes. (B) To further elucidate the functional roles of these genes, KEGG functional enrichment analysis was performed, and these genes were primarily involved in cell division and the cell cycle, with a regulatory effect on chromosome functionality and kinematics. (C) The correlation heatmap shows that these genes are strongly correlated. (D) Multivariate Venn diagram methods were utilized to identify key genes critical to the above pathways.

As shown in **Figure 2A**, BORA, CDCA4, CDCA5, CDK1, CDKN3, CENPH, CIP2A, DSCC1, FAM83D, KIF11, KIF20A, KIF2C, KNL1, MCM10, NEK2, SGO2, SHCBP1, SKA3, and UHRF1 are interconnected in the protein-protein interaction networks (PPI). By utilizing the online application DAVID Bioinformatics Resources to analyze their functional enrichment of these genes, we observed that these genes are predominantly involved in chromosome separation, cell cycle and cell division (**Figure 2B** and **Table S2**). Meanwhile, these genes show strong correlations (**Figure 2C**). Furthermore, we found that SGO2, KIF2C, NEK2, and KNL1 play significant roles in these processes (**Figure 2D**).

### SGO2 increases the risk of poor prognosis for LUAD

**Figure 3:**
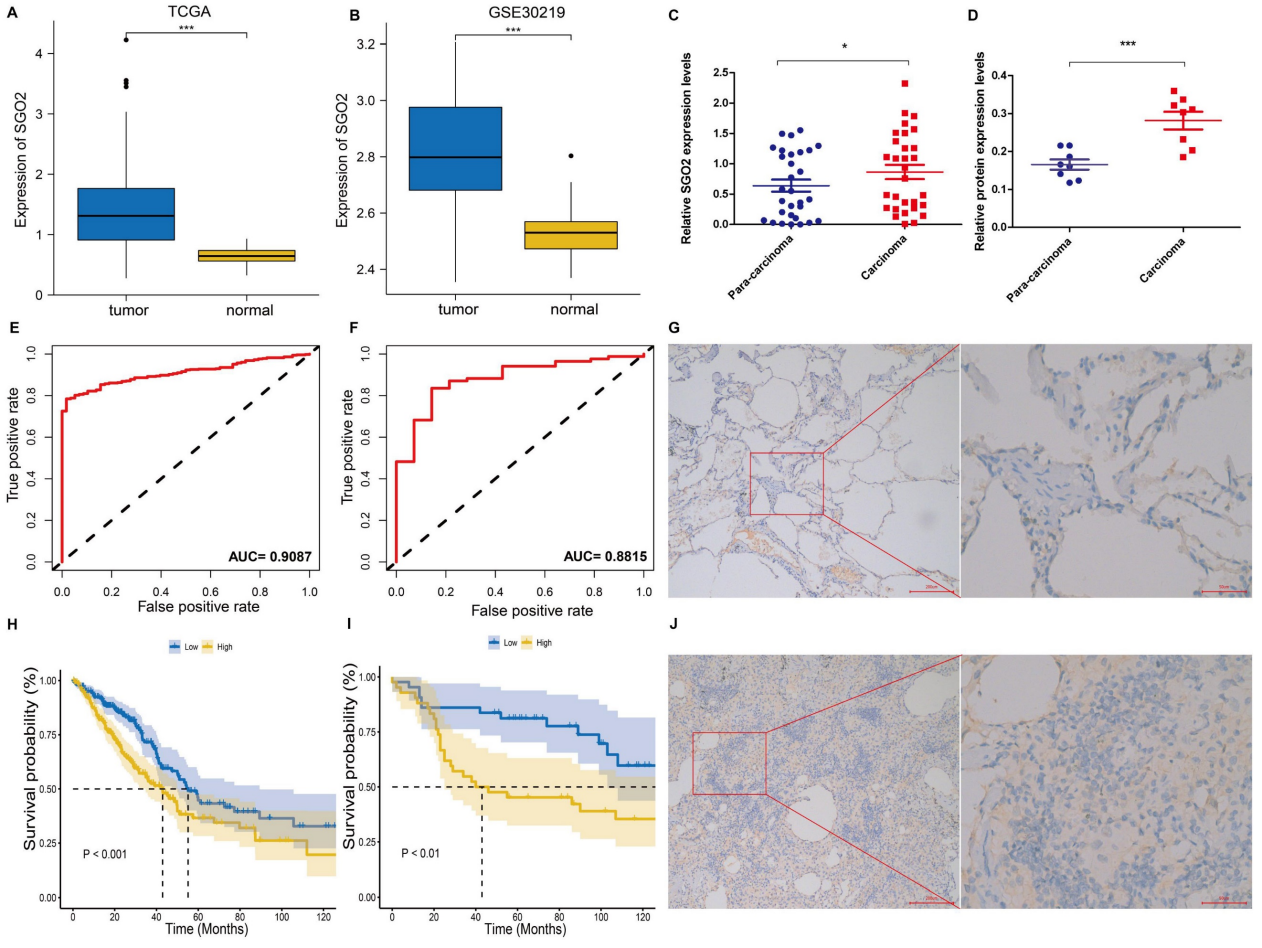
** SGO2 increases the risk of poor prognosis for LUAD.** (A, E, and H) Based on the TCGA database, SGO2 expression, ROC curve, and survival analysis in LUAD. (B, F, and I) Based on the GEO database, the validation dataset GSE30219 was used to verify the SGO2 expression, ROC curve, and survival analysis in LUAD. (C) The expression of SGO2 in LUAD and normal tissue. (D) The protein expression of SGO2 in LUAD and normal tissue. (G and J) Immunohistochemical staining of SGO2 in LUAD and normal tissue.

TCGA data analysis exhibited a higher expression of SGO2 in LUAD as compared to normal lung tissues (**Figure 3A**). The ROC curve was employed to differentiate between LUAD and normal lung tissue, with an area under the curve of 0.9087, indicating a high diagnostic value of SGO2 (**Figure 3E**). Further, K-M curve analysis indicated a negative impact of high SGO2 expression on LUAD prognosis (**Figure 3H**). Notably, the validation dataset GSE30219 also shows overexpression of SGO2 in LUAD (**Figure 3B**), and with an area under the ROC curve of 0.8815, and K-M curve analysis indicates a negative correlation between SGO2 and OS (**Figure 3F and Figure 3I**). Additionally, according to qRT-PCR in **Figure 3C**, the expression of SGO2 in tumors (0.864±0.642) was higher than that in normal tissues (0.639±0.543, P=0.025). At the same time, we also measured SGO2 protein expression, and in **Figure 3D** show that SGO2 protein expression in carcinoma (0.2817±0.065) was significantly higher than that in para-carcinoma (0.1658±0.038, P=0.001). Finally, IHC staining suggests deeper staining intensity of carcinoma compared to para-carcinoma (**Figure 3G and Figure 3J**). This evidence highlights SGO2 as a potential biomarker for predicting prognosis in LUAD.

### High SGO2 is associated with higher TNM stage of LUAD

**Figure 4:**
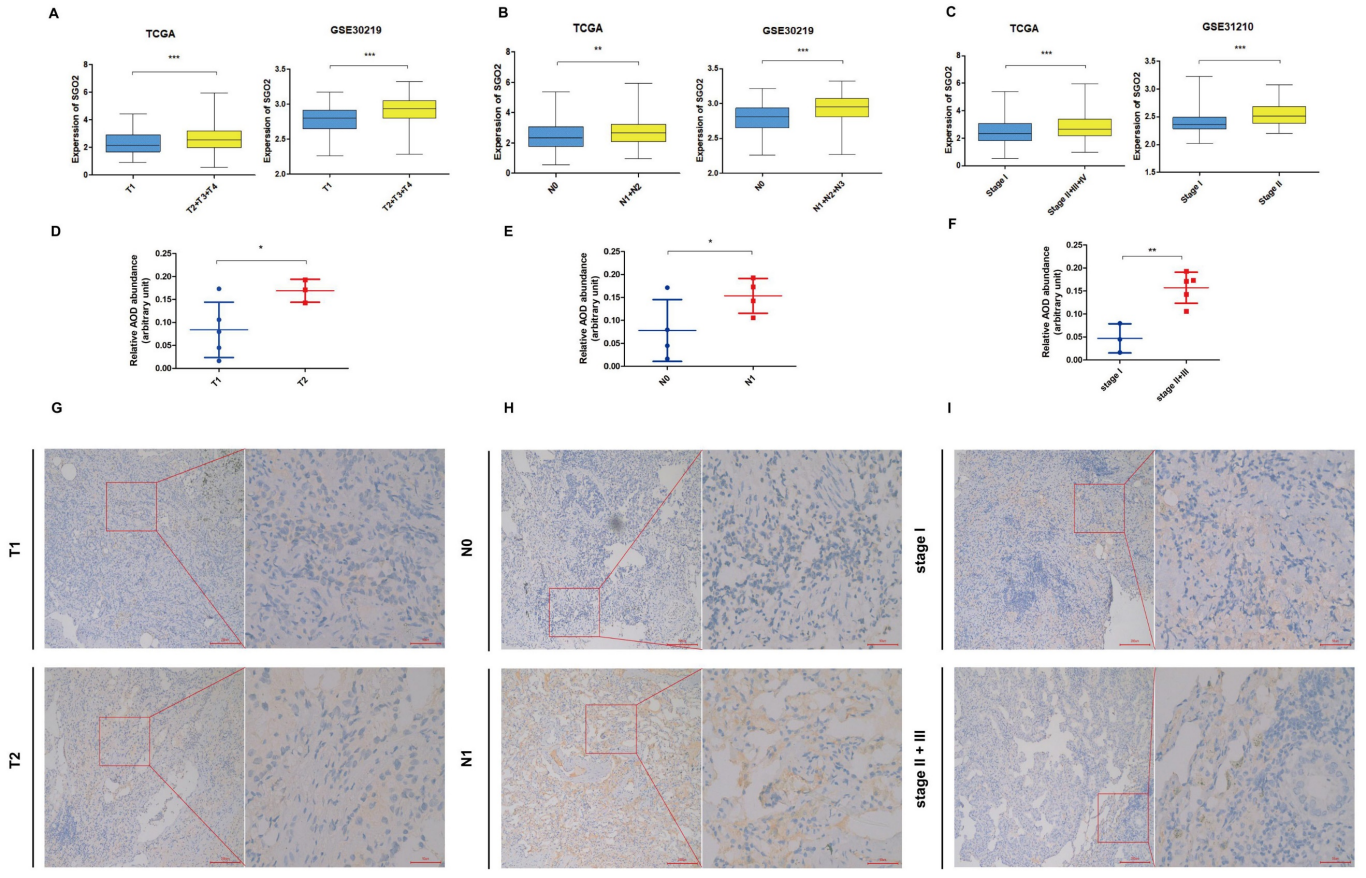
** High SGO2 is associated with a higher TNM stage of LUAD.** (A) Based on the TCGA and GSE30219, the expression of SGO2 in T1 vs T2. (B) Based on the TCGA and GSE30219, the expression of SGO2 in N0 vs N1. (C) Based on the TCGA and GSE31210, the expression of SGO2 in stage I vs stage II + III. (D and G) Based on IHC, the protein expression and staining of SGO2 in T1 vs T2. (E and H) Based on IHC, the protein expression and staining of SGO2 in N0 vs N1. (F and I) Based on IHC, the protein expression and staining of SGO2 in stage I vs stage II + III.

The TCGA dataset analysis demonstrated that high expression of SGO2 in LUAD was significantly associated with higher T, N, and stage. The findings were further confirmed using the validation dataset GSE30219 and GSE31210 (**Figure 4A-C**). The results of IHC were shown in **Figure 4D-F**, and the protein expression of SGO2 was also associated with higher T, N, and stage (T1: T2 = 0.084± 0.06: 0.17±0.03, p = 0.031; N0:N1=0.078±0.07:0.154±0.04, p = 0.05; stage I:stage III=0.047±0.03:0.157±0.03, p=0.002). Meanwhile, LUAD with higher TNM stage has deeper staining intensity and density (**Figure 4G-I**).

### SGO2 silencing inhibits proliferation of lung cancer cells

**Figure 5:**
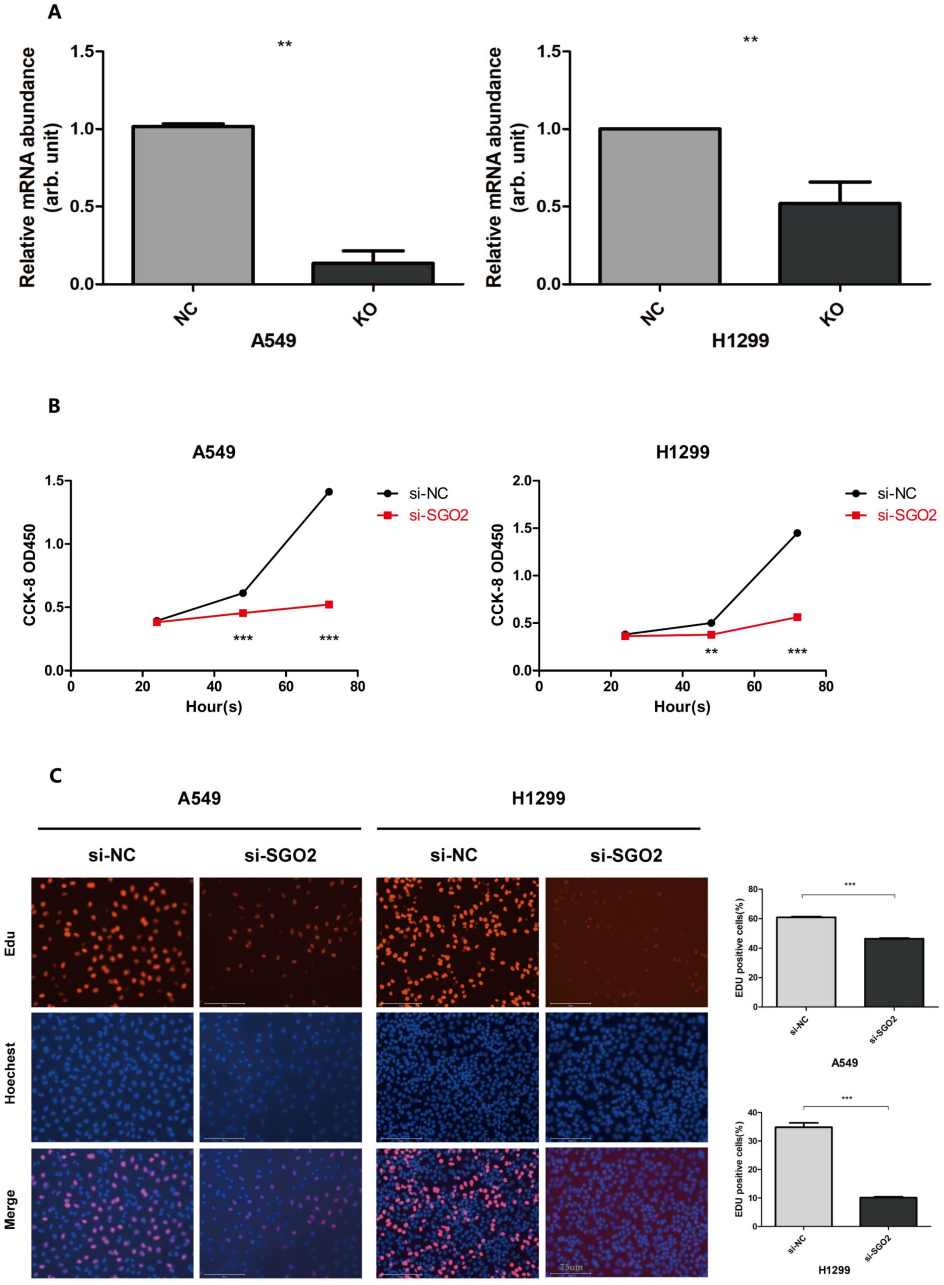
** SGO2 silencing inhibits the proliferation of lung cancer cells.** (A)SGO2 expression after knockdown in A549 and H1299. (B)CCK8 assays were performed to detect A549 and H1299 proliferation. (C)Edu assays were performed to detect A549 and H1299 proliferation.

To confirm the role of SGO2 in lung cancer cell proliferation, we employed siRNA targeting SGO2 to transfect A549 and H1299 cells. RT-qPCR analysis was then performed to analyze the mRNA expression levels of SGO2(**Figure 5A**). Subsequently, we carried out EdU and CCK8 assays on the normal control (NC) and SGO2 interference (SI) groups to evaluate SGO2's effect on cell growth. Our CCK8 assay showed that SGO2 knockdown reduced cell proliferation within 48 h and reached a maximum at 72 h (**Figure 5B**). Moreover, the EdU assay revealed a significant reduction in cell growth, thus underscoring SGO2's contribution to lung cancer cell proliferation (**Figure 5C**).

### The downregulation of SGO2 affects the migration and invasion of lung cancer cells

**Figure 6:**
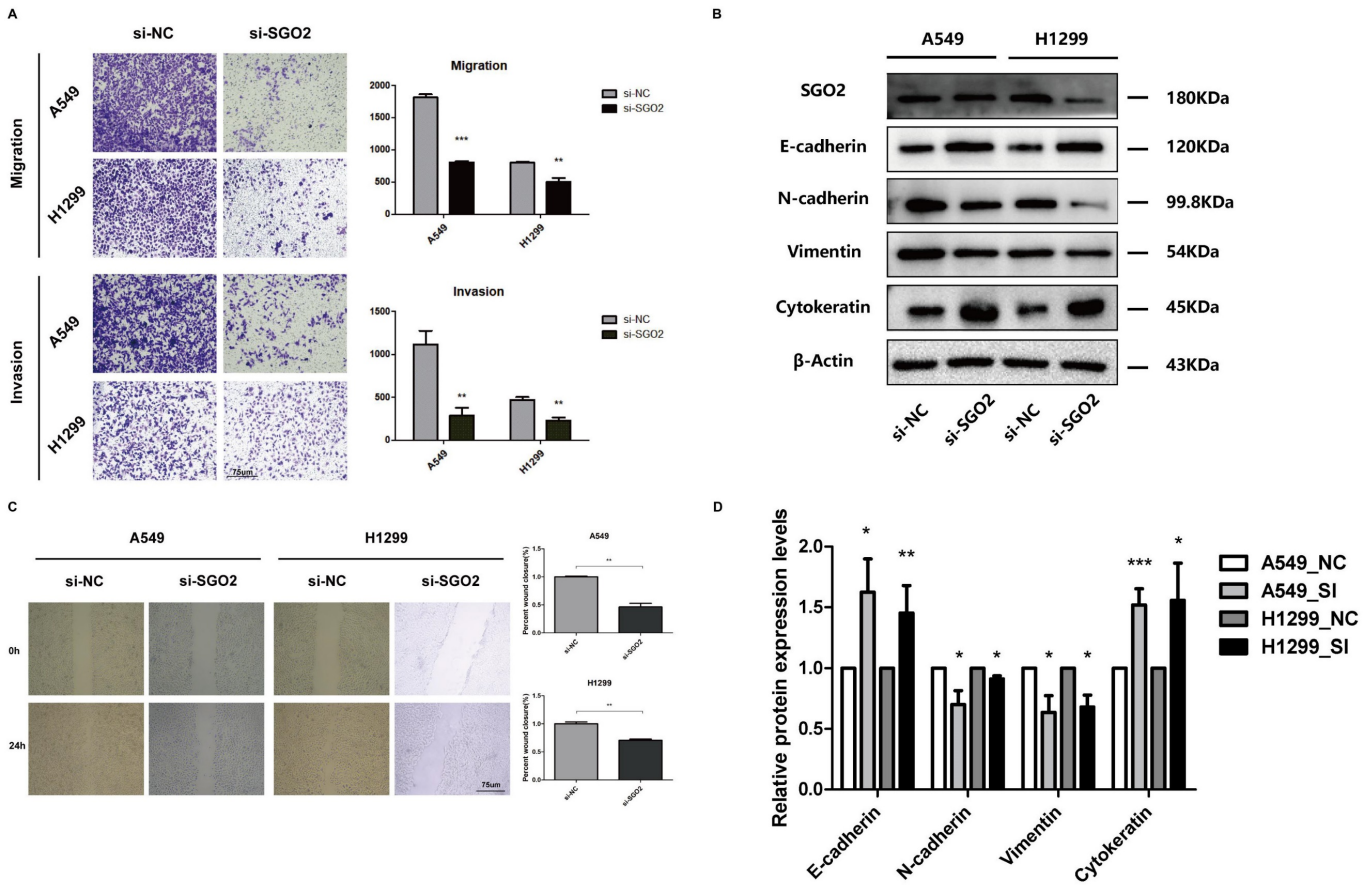
** The downregulation of SGO2 affects migration, invasion, and EMT.** (A) A Transwell assay was performed to examine the effect of SGO2 knockdown on A549 and H1299 cells. (C) A wound healing assay was performed to examine the effect of SGO2 knockdown on A549 and H1299 cells. (B and D) WB assay the expression levels of E-cadherin, N-cadherin, Vimentin, and Cytokeratin in A549 and H1299 to evaluate the effect on EMT after SGO2 knockdown.

We further investigated the impact of SGO2 on lung cancer cell migration using both wound healing and migration assays. Our findings revealed that siRNA-mediated knockdown of SGO2 significantly reduced cell migration ability in both A549 and H1299 cells when compared to the normal control (NC) group (**Figure 6A**). In addition, we utilized Transwell assays to measure lung cancer cell migration and invasion and demonstrated that SGO2 knockdown significantly suppressed cell migration and invasion (**Figure 6C**).

### SGO2 plays an important role in the EMT process of lung cancer cells

We performed Western blot analysis to assess the protein levels of EMT-related markers in A549 and H1299. Remarkably, inhibiting SGO2 increased the protein levels of essential epithelial markers, such as E-cadherin and cytokeratin, while reducing the expression of critical mesenchymal markers, including Vimentin and N-cadherin (**Figure 6B and Figure 6D**). These results suggest that LUAD acquires the ability to transition from epithelial to mesenchymal by increasing SGO2 expression, and then to distant metastasis.

### SGO2 expression and tumor immune infiltration

**Figure 7:**
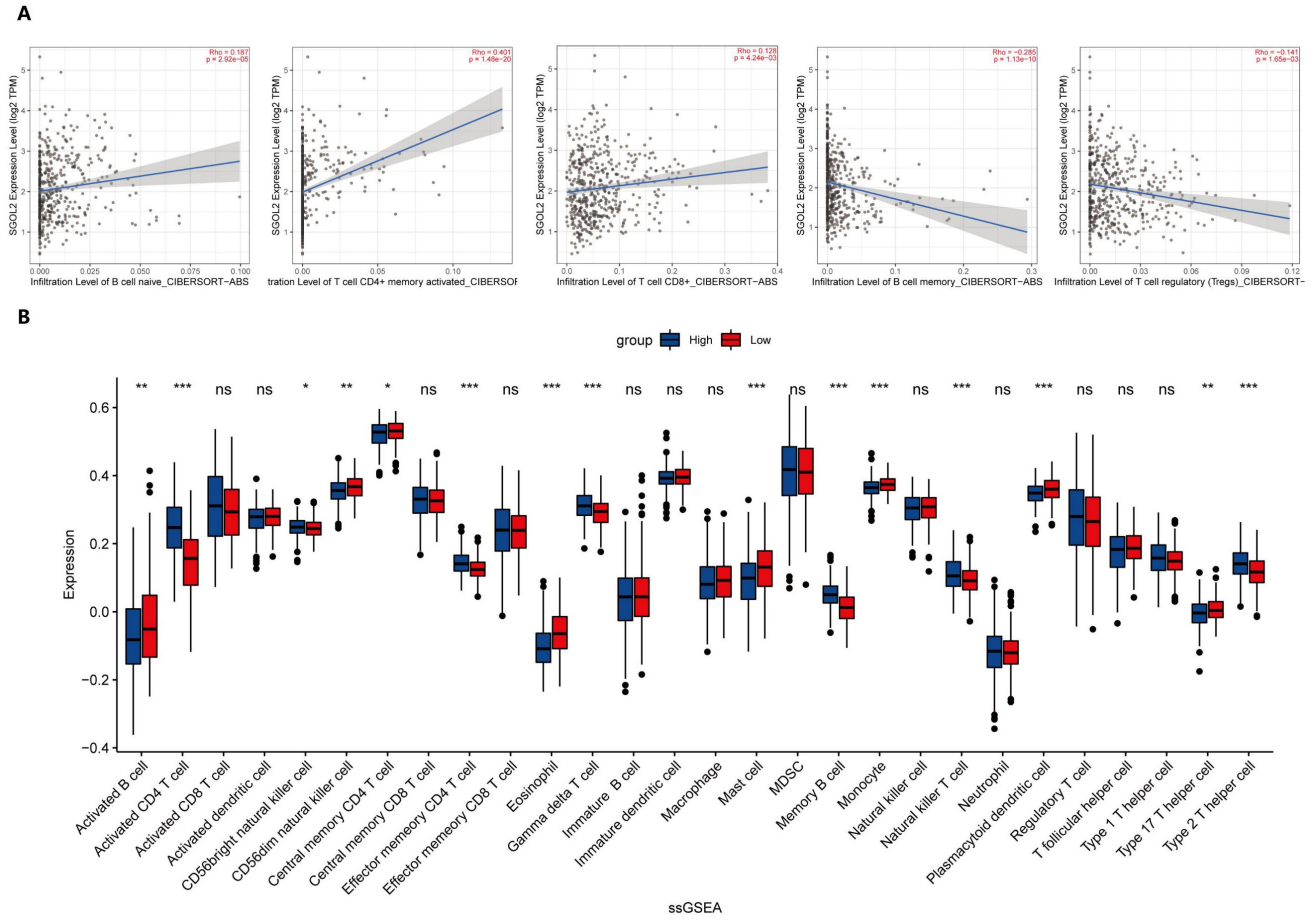
** SGO2 expression and tumor immune infiltration.** (A) the expression of SGO2 was positively correlated with the infiltration of Memory B cells, Activated CD4+ memory T cells, and CD8+ T cells, while the infiltration of Memory B cells and Tregs decreased with increasing SGO2 expression. (B) Correlation of SGO2 expression with 28 distinct types of tumor-infiltrating immune cells based on ssGSEA.

The CIBERSORT ABS.MODE algorithm was used to determine the infiltration of nine tumor-infiltrating lymphocytes (TILs) in LUAD. The results indicated that the expression of SGO2 was positively correlated with the infiltration of Memory B cells, Activated CD4+ memory T cells, and CD8+ T cells, while the infiltration of Memory B cells and Tregs decreased with increasing SGO2 expression (**Figure 7A**). Single-sample gene set enrichment analysis (ssGSEA) provided other results showing that the expression of SGO2 was positively correlated with the infiltration of seven TIIC subtypes, including Activated CD4+ T cell, CD56bright natural killer cell, Effector memory CD4+ T cell, Gamma delta T cell, Memory B cell, Natural killer T cell, and Type 2 T helper cell. In contrast, SGO2 was negatively correlated with the infiltration of eleven TIIC subtypes, including Activated B cell, CD56dim natural killer cell, Central memory CD4+ T cell, Eosinophil, Mast cell, Monocyte, Plasmacytoid dendritic cell, and type 17 T helper cells (**Figure 7B**).

### Evaluation of therapeutic sensitivity for high SGO2 expression

**Figure 8:**
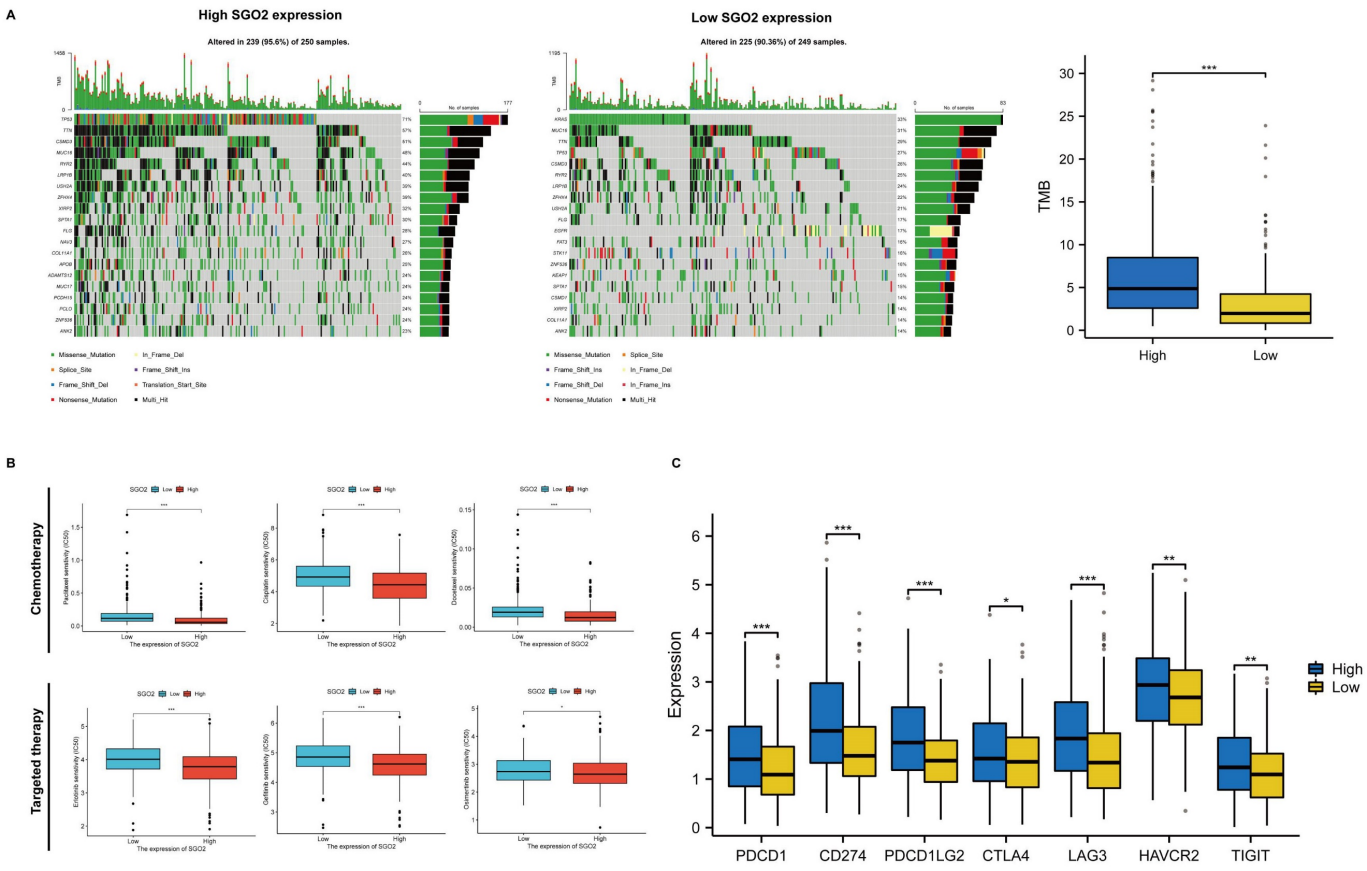
** High SGO2 has multiple therapeutic benefits.** (A) Comparison of mutational landscapes of SGO between high cluster and low cluster, and Comparison of tumor mutation burden (TMB) between two clusters. (B) Comparison of first-line chemotherapy and targeted therapy drug targets of high and low SGO2 clusters. (C) Comparison of immunomodulatory drug targets of high and low SGO2 clusters.

We evaluated the mutational conditions of LUAD to understand the impact of different gene mutations on treatment efficacy, and more gene mutations occurred in the high SGO2 cluster compared with the low SGO2 cluster (**Figure 8A**). In addition, we also evaluated the efficacy of first-line chemotherapy and targeted therapy in LUAD, including Paclitaxel, Cisplatin, Docetaxel, Erlotinib, Gefitinib, and Osimertinib. Compared with the low SGO2 cluster, the high SGO2 cluster had a lower half-inhibitory concentration (IC50), indicating a better therapeutic effect of high SGO2 expression cluster (**Figure 8B**). Finally, we compared the expression of immunomodulatory targets between the two clusters and found that the major regulatory targets (PD1, PDL1, PDL2, CTLA4, LAG3, HAVCR2, and TIGIT) were significantly more expressed in the high SGO2 cluster (**Figure 8C**). These results suggest that high SGO2 cluster may respond better to chemotherapy, targeted therapy as well as immunotherapy than low SGO2 cluster.

## Discussion

As detection technologies continue to expand, early cancer screening is becoming more commonplace, leading to an increasing number of non-small cell lung cancers identified at earlier stages. Despite early intervention, however, some patients with lung adenocarcinoma still experience recurrence and unfavorable outcomes.^1^ To unravel the mechanisms driving these outcomes, we employed bioinformatics techniques to identify differentially expressed genes that correlated with lower overall survival and TNM stage classification. Our bioinformatics analyses revealed the critical role that these genes play in regulating cell cycle and cell division by analyzing protein interaction networks and functional enrichment. We are more concerned with the impact of accurate chromosomal separation on cancer, and Klaasen SJ et al. showed that nuclear chromosome locations located not at the center of the nucleus increase chromosome separation errors and lead to aneuploidy and micronuclei production, which may affect the dynamics of recurring aneuploidy and genomic rearrangement patterns seen in cancer, thereby affecting tumor growth, metastasis, and relapse.^22^ The Shugoshin protein protects nuclear chromosomes to accurately line up and separate on cell plates in the middle of cell division.^23^ Remarkably, we uncovered a previously unrecognized role for SGO2 in LUAD and designed experiments to investigate its effects on vital aspects of cancer progression, such as proliferation, migration, invasion, and EMT. Consequently, we are building upon these discoveries to deepen our understanding of LUAD and ultimately improve patient outcomes.

Chromosomal instability (CIN) resulting from genetic mutations or environmental factors such as smoking is believed to be responsible for aneuploidy, chromosome breaks, DNA damage, and/or whole chromosome gain/loss, which can contribute to the development of numerous cancers, including lung cancer.^24^ Therefore, the study of Shugoshin proteins, particularly SGO1, which regulates the accurate isolation of sister chromatids during mitosis, has attracted considerable interest.^25^ Initially, research focused on the role of SGO1 in mitigating CIN-induced cancer, but as the scope of the study expanded, it was discovered that overexpression of shear variants SGO1-P1 and SGO1-B1 in tumor cell lines induces abnormal is separation, premature separation of chromatids, and delayed mitotic processes, ultimately leading to increased taxanes resistance.^26^, ^27^ Further studies verified the cancer-promoting effect of SGO1 through experiments. Liu et al. concluded that SGO1 inhibits the growth of lung cancer cells and promotes apoptosis.^28^ Fei et al. proved that SGO1 is highly expressed in HCC tissues, is a biomarker with poor prognosis, may be related to immune cell infiltration in HCC, and may enhance the proliferation, invasion, and migration of HCC cells.^29^ Thus, SGO1 may offer a new therapeutic avenue for cancer treatment. As a result, the study of SGO2, a homolog of SGO1, has gained importance in recent years. Initial research focused on the role of SGO2 in protecting centromeric cohesion during mammalian meiosis I, but subsequent studies found that high SGO2 expression is associated with poor prognosis in a variety of cancers, including liver cancer, prostate cancer, and glioma.^15^, ^30^-^33^ By using cancer stem cell-associated genes (RAB10, TCOF1, and PSMD14), Liang et al. constructed a prognostic model for HCC and identified SGO2 as a potential therapeutic target.^34^ Deng et al. used bioinformatics to verify the association between high SGO2 expression and poor overall survival and advanced clinicopathological features in HCC.^31^ Additionally, Jiang and Li et al.'s investigation into the regulation of abnormal cell division by cell cycle and cell division-related proteins in HCC indirectly revealed the therapeutic potential of SGO2.^35^, ^36^ These findings provide evidence that SGO2 regulates cancer development and offer a crucial foundation for the development of targeted therapies.

In the present study, we have utilized the TCGA and GEO databases to corroborate that elevated levels of SGO2 expression in lung adenocarcinoma are associated with a higher TNM stage and a poorer prognosis. Additionally, we analyzed the expression profile of SGO2 and its protein in both cancerous and normal lung tissues, revealing a significant increase in SGO2 expression within cancer tissues and a stronger staining intensity. Remarkably, we observed a correlation between SGO2 intensity levels and TNM stage, echoing earlier findings by Kao Y et al. on SGO2's association with glioma grade, and its heightened expression driving an unfavorable prognosis.^15^ Our hypothesis is that this may stem from amplified proliferative capacity in higher-grade tumors. To investigate this, we employed siRNA to interfere with SGO2 expression in lung cancer cells. We found that SGO2 knockdown significantly inhibited cell proliferation, migration, invasion and EMT transition behavior. Therefore, our results imply that SGO2, as a regulator of the cell cycle, promotes cell proliferation and may augment the metastatic ability of tumor cells, underscoring its prospective use as a new prognostic biomarker for LUAD. In previous research, SGO2 has demonstrated its role in facilitating the separation of sister chromatids during cell division. By binding to Mad2, it forms an inhibitory complex, like Securin, that competitively binds to isomorphs. Interestingly, the competitive inhibition of Sgo2 does not impact its binding to protein phosphatase 2 (PP2A), which dephosphorylates centromere and strengthens centromere cohesion.^13^, ^23^, ^37^ In addition, during mitosis, SGO2 acts as a scaffold protein for the chromosomal passenger complex (CPC), sustaining the arrangement of centromeres in the equatorial plane and promoting cell division.^38^, ^39^

The tumor microenvironment is a key factor in the development and progression of tumors. In this study, we aimed to investigate the relationship between SGO2 and tumor immune invasion in LUAD. Our findings revealed that high levels of SGO2 expression in LUAD were associated with increased infiltration of CD4+ T cells, which are known to play a critical role in anti-tumor responses by enhancing the anti-tumor activity of immune cells and producing cytokines like TNFα and IFN-γ.^40^ Functionally, CD4+ helper T cells can be divided into different subsets, including Th1, Th2, Th17, and regulatory T cells (Tregs), based on their cytokine secretion profiles.^41^ Our results revealed that Th2 cell tumor infiltration increased while Th17 cell tumor infiltration decreased. Th2 cells secrete IL10 and inhibit inflammation, which can promote tumor development, whereas Th17 cells indirectly participate in anti-tumor effects by promoting T cell recruitment to tumors and CD8+ T cell activation.^41^, ^42^ Confusingly, we observed opposing trends in the infiltration of memory B cells using the two algorithms. Memory B cells are key players in adaptive immunity and can proliferate and differentiate into plasma cells when stimulated by T effector cells or strong BCR stimulation.^43^ Previous research has shown that B cells can induce anti-tumor immunity and produce antibodies by acting as antigen-presenting cells.^44^ However, our findings indicated a decrease in the infiltration of monocyte, dendritic cell, and mast cell infiltrates involved in anti-tumor and antigen presentation. While monocytes have diverse roles at various stages of cancer, including both anti-tumor and tumor-promoting effects, DCs enhance anti-tumor T cell immunity by presenting tumor antigens.^45^, ^46^ Mast cells have been shown to have an anti-tumor role in lung cancer but may be dependent on the characteristics of the TME.^47^ In summary, the TME is a complex milieu that guides immune cells to play either anti-tumor or tumor-promoting effects. Evaluating the role of immune cells on tumor growth through the proportion of immune cells with antagonistic functions in TME has become a popular approach. Our study used CIBERSORT ABS. MODE and ssGSEA analysis to show that high expression of SGO2 in LUAD was associated with a low level of infiltration of major anti-tumor immune cells, indicating that SGO2 overexpression may hinder anti-tumor immunity and lead to a poorer prognosis. Thus, our study highlights SGO2's prognostic value in guiding LUAD therapy.

In patients with advanced LUAD presenting positive driver genes without drug resistance mutations, targeted therapy is the preferred first-line treatment. Conversely, for advanced LUAD cases with negative driver genes, platinum combined with pemetrexed, paclitaxel, or docetaxel is the chosen first-line therapy. Prevalent LUAD driver genes encompass EGFR-sensitive mutations, ALK fusion genes, ROS1 fusion genes, and BRAF V600 mutations. For patients with EGFR-susceptible mutations, EGFR-TKIs such as Osimertinib, Gefitinib, and Erlotinib are advised, while Crizotinib is recommended for those positive for ALK fusion and ROS1 fusion genes. Additionally, Dabrafenib plus Trametinib is suggested for patients with advanced NSCLC and positive BRAF V600 mutations.^48^ The utilization of immune checkpoint inhibitors (ICIs) in advanced LUAD can prolong survival for select lung cancer patients, offering an innovative and effective treatment.^49^ Regarding gene mutation analysis, high SGO2 clusters exhibit a higher tumor mutational burden (TMB) compared to low SGO2 clusters. Furthermore, TP53, a tumor suppressor gene and the most frequently mutated gene in human cancer, displays a 71% mutation rate within high SGO2 clusters.^50^ Although there is no recommended medication available for TP53 mutations, our study demonstrates that high SGO2 clusters are more responsive to chemotherapy and targeted therapies, with lower IC50 values for Paclitaxel, Cisplatin, Docetaxel, Erlotinib, Gefitinib, and Osimertinib. TMB may affect patients' responses to ICIs by modulating the production of immunogenic peptides. A significant correlation between high TMB and ICI treatment has been observed across various tumors.^51^ In LUAD, this conclusion is that the high SGO2 cluster has higher TMB and expression levels of 7 immune checkpoints (PDCD1, CD274, PDCD1LG2, CTLA4, LAG3, HAVCR2, and TIGIT), consistent with previous findings, which show lower immune cell infiltration and attenuated anti-tumor responses could promote tumor growth and migration. Therefore, we believe that high SGO2 expression is strongly associated with the increasing of immune evasion and tolerance. The interaction of PDCD1 (PD-1) with CD274 (PD-L1) can impair the immune environment, reducing T cell activity and inducing T cell depletion alongside decreased cytokine levels, such as TNFα and IFN-γ.^52^ This allows tumor cells to escape immune surveillance. PDCD1LG2 (PD-L2) serves as another PDCD1 ligand, inhibiting T cell activation and enabling tumor cells to evade immune responses.^53^ CTLA-4 is a critical mediator in T cell activation and tolerance, regulating Treg-mediated immunosuppression and acting as a feedback control mechanism upon resting T cell activation.^54^ LAG3 can diminish T cell proliferation and cytokine secretion, leading to CD8+ T cell depletion and failures in antitumor immunity.^55^ HAVCR2 (TIM-3) acts as a negative immune checkpoint, suppressing antitumor immunity by inducing T cell depletion.^56^ TIGIT can impair antitumor immune responses by causing T cell and natural killer cell dysfunction.^57^ Combining anti-PD-1/anti-LAG3 or anti-PD-1/anti-TIGIT immunotherapy may yield better inhibitory effects on tumor growth compared to monotherapy.^57^, ^58^ In addition, Koyama et al. have reported that TIM-3 expression increases upon the development of anti-PD-1 adaptive resistance.^59^ This suggests that high SGO2 clusters might be more suitable for immunotherapy to restore immune function and achieve better treatment outcomes.

## Conclusions

In summary, this study utilized bioinformatics analysis to elucidate the relationship between SGO2 and LUAD. Our results indicate that SGO2 is highly expressed in LUAD and positively correlated with TNM stage, suggesting its potential utility in predicting early metastasis and unfavorable outcomes. For another, downregulation of SGO2 inhibited proliferation, migration, invasion, and EMT of lung cancer cells, underscoring the oncogenic role of dysregulated SGO2 in promoting cancer development through the stabilization of mitotic centromeric and isolation of sister chromatids. Notably, high SGO2 expression may have poorer anti-tumor immunity and may therefore be more suitable for immunotherapy to re-establish immune function. These findings imply that SGO2 offer significant diagnostic and therapeutic potential as a prognostic marker and therapeutic target for LUAD patients.

## Supplementary Material

Supplementary tables.

## Figures and Tables

**Table 1:** Clinical information of LUAD parents

	event = 0	event = 1	p.value
**Gender**			0.767
Female	194(40.4%)	68(14.2%)	
Male	164(34.2%)	54(11.2%)	
**Age**			0.989
<60	100(40.4%)	34(7.1%)	
≥60	258(53.8%)	88(18.3%)	
**Stage**			<0.001
Stage I	219(40.4%)	40(8.3%)	
Stage II+III+IV	139(29%)	82(17.1%)	
**T**			<0.001
T1	140(29.1%)	23(4.8%)	
T2 + T3 + T4	218(45.4%)	99(20.6%)	
**N**			<0.001
N0	266(55.4%)	54(11.3%)	
N1 + N2	92(19.1%)	68(14.2%)	
**M**			0.019
M0	345(71.2%)	111(23.1%)	
M1	13(2.7%)	11(2.3%)	

*event = 0: Alive, event = 1: Dead 1. The table 1 contains clinical information on 480 LUAD patients 2. All data are graded data, with binary classification of continuous variables

## References

[1] Siegel RL, Miller KD, Wagle NS, Jemal A (2023). Cancer statistics, 2023. Ca-Cancer J Clin.

[2] Nurwidya F, Murakami A, Takahashi F, Takahashi K (2012). Lung cancer stem cells: Tumor biology and clinical implications. Asia-Pac J Clin Onco.

[3] Ou SHI, Zell JA, Ziogas A, Anton-Culver H (2007). Prognostic factors for survival of stage I nonsmall cell lung cancer patients - A population-based analysis of 19,702 stage I patients in the California Cancer Registry from 1989 to 2003. Cancer-Am Cancer Soc.

[4] Yao YX, Dai W (2012). Shugoshins function as a guardian for chromosomal stability in nuclear division. Cell Cycle.

[5] Kawashima SA, Tsukahara T, Langegger M, Hauf S, Kitajima TS, Watanabe Y (2007). Shugoshin enables tension-generating attachment of kinetochores by loading Aurora to centromeres. Gene Dev.

[6] Kanoh J (2017). Unexpected roles of a shugoshin protein at subtelomeres. Genes Genet Syst.

[7] Takii R, Fujimoto M, Matsumoto M, Srivastava P, Katiyar A, Nakayama KI (2019). The pericentromeric protein shugoshin 2 cooperates with HSF1 in heat shock response and RNA Pol II recruitment. Embo J.

[8] Love MI, Huber W, Anders S (2014). Moderated estimation of fold change and dispersion for RNA-seq data with DESeq2. Genome Biol.

[9] Terry M (2000). Therneau PMG. Modeling Survival Data: Extending the Cox Model: Springer, New York.

[10] Biecek AKaMKaP survminer: Drawing Survival Curves using 'ggplot2'. 2021.

[11] Sherman BT, Hao M, Qiu J, Jiao XL, Baseler MW, Lane HC (2022). DAVID: a web server for functional enrichment analysis and functional annotation of gene lists (2021 update). Nucleic Acids Res.

[12] R C, Team (2022) R: A language and environment for statistical computing. R Foundation for Statistical Computing, Vienna, Austria. URL https://www.R-project.org/.

[13] Hellmuth S, Gomez-H L, Pendas AM, Stemmann O (2020). Securin-independent regulation of separase by checkpoint-induced shugoshin-MAD2. Nature.

[14] Livak KJ, Schmittgen TD (2001). Analysis of relative gene expression data using real-time quantitative PCR and the 2(T)(-Delta Delta C) method. Methods.

[15] Kao Y, Tsai WC, Chen SH, Hsu SY, Huang LC, Chang CJ (2021). Shugosin 2 is a biomarker for pathological grading and survival prediction in patients with gliomas. Sci Rep-Uk.

[16] Gantchev J, Villarreal AM, Xie PX, Lefrancois P, Gunn S, Netchiporouk E (2019). The Ectopic Expression of Meiosis Regulatory Genes in Cutaneous T-Cell Lymphomas (CTCL). Front Oncol.

[17] Schneider CA, Rasband WS, Eliceiri KW (2012). NIH Image to ImageJ: 25 years of image analysis. Nat Methods.

[18] Li TW, Fu JX, Zeng ZX, Cohen D, Li J, Chen QM (2020). TIMER2.0 for analysis of tumor-infiltrating immune cells. Nucleic Acids Res.

[19] Hanzelmann S, Castelo R, Guinney J (2013). GSVA: gene set variation analysis for microarray and RNA-Seq data. Bmc Bioinformatics.

[20] Mayakonda A, Lin DC, Assenov Y, Plass C, Koeffler HP (2018). Maftools: efficient and comprehensive analysis of somatic variants in cancer. Genome Res.

[21] Maeser D, Gruener RF, Huang RS (2021). oncoPredict: an R package for predicting in vivo or cancer patient drug response and biomarkers from cell line screening data. Brief Bioinform.

[22] Klaasen SJ, Truong MA, van Jaarsveld RH, Koprivec I, Stimac V, de Vries SG (2022). Nuclear chromosome locations dictate segregation error frequencies. Nature.

[23] Yu HT (2011). A mad partner for Shugoshin in meiosis. Embo J.

[24] Yamada HY, Kumar G, Zhang Y, Rubin E, Lightfoot S, Dai W (2016). Systemic chromosome instability in Shugoshin-1 mice resulted in compromised glutathione pathway, activation of Wnt signaling and defects in immune system in the lung. Oncogenesis.

[25] Zhang Q, Liu H (2020). Functioning mechanisms of Shugoshin-1 in centromeric cohesion during mitosis. Essays Biochem.

[26] Matsuura S, Kahyo T, Shinmura K, Iwaizumi M, Yamada H, Funai K (2013). SGOL1 variant B induces abnormal mitosis and resistance to taxane in non-small cell lung cancers. Sci Rep.

[27] Kahyo T, Iwaizumi M, Shinmura K, Matsuura S, Nakamura T, Watanabe Y (2011). A novel tumor-derived SGOL1 variant causes abnormal mitosis and unstable chromatid cohesion. Oncogene.

[28] Liu L, Zhang N, Liu J, Min J, Ma N, Liu N (2012). Lentivirus-mediated siRNA interference targeting SGO-1 inhibits human NSCLC cell growth. Tumour Biol.

[29] Fei X, Liu S, Liu P, Wang X, Zhu C, Hou J (2022). Identification and validation of a potential key gene SGOL1 for poor prognosis in hepatocellular carcinoma based on a bioinformatics approach. Front Oncol.

[30] Llano E, Gomez R, Gutierrez-Caballero C, Herran Y, Sanchez-Martin M, Vazquez-Quinones L (2008). Shugoshin-2 is essential for the completion of meiosis but not for mitotic cell division in mice. Genes Dev.

[31] Deng M, Li S, Mei J, Lin W, Zou J, Wei W (2021). High SGO2 Expression Predicts Poor Overall Survival: A Potential Therapeutic Target for Hepatocellular Carcinoma. Genes (Basel).

[32] Lv T, He D, Zhang X, Guo X, Li Z, Zhang A (2022). SGOL2 promotes prostate cancer progression by inhibiting RAB1A ubiquitination. Aging (Albany NY).

[33] Hu Q, Liu Q, Zhao Y, Zhang L, Li L (2022). SGOL2 is a novel prognostic marker and fosters disease progression via a MAD2-mediated pathway in hepatocellular carcinoma. Biomark Res.

[34] Liang XY, Zhang Y, He YN, Liu XY, Ding ZH, Zhang XD (2022). A cancer stem cell associated gene signature for predicting overall survival of hepatocellular carcinoma. Front Genet.

[35] Jiang SS, Ke SJ, Ke ZL, Li J, Li X, Xie XW (2021). Cell Division Cycle Associated Genes as Diagnostic and Prognostic Biomarkers in Hepatocellular Carcinoma. Front Mol Biosci.

[36] Li L, Huang K, Zhao H, Chen B, Ye Q, Yue J (2020). CDK1-PLK1/SGOL2/ANLN pathway mediating abnormal cell division in cell cycle may be a critical process in hepatocellular carcinoma. Cell Cycle.

[37] Meadows JC, Lancaster TC, Buttrick GJ, Sochaj AM, Messin LJ, Mora-Santos MD (2017). Identification of a Sgo2-Dependent but Mad2-Independent Pathway Controlling Anaphase Onset in Fission Yeast. Cell Rep.

[38] Asai Y, Fukuchi K, Tanno Y, Koitabashi-Kiyozuka S, Kiyozuka T, Noda Y (2019). Aurora B kinase activity is regulated by SET/TAF1 on Sgo2 at the inner centromere. J Cell Biol.

[39] Asai Y, Matsumura R, Hasumi Y, Susumu H, Nagata K, Watanabe Y (2020). SET/TAF1 forms a distance-dependent feedback loop with Aurora B and Bub1 as a tension sensor at centromeres. Sci Rep-Uk.

[40] Tay RE, Richardson EK, Toh HC (2021). Revisiting the role of CD4(+) T cells in cancer immunotherapy-new insights into old paradigms. Cancer Gene Ther.

[41] Duan MC, Zhong XN, Liu GN, Wei JR (2014). The Treg/Th17 paradigm in lung cancer. J Immunol Res.

[42] Williams LM, Ricchetti G, Sarma U, Smallie T, Foxwell BM (2004). Interleukin-10 suppression of myeloid cell activation-a continuing puzzle. Immunology.

[43] Seifert M, Kuppers R (2016). Human memory B cells. Leukemia.

[44] Wennhold K, Shimabukuro-Vornhagen A, von Bergwelt-Baildon M (2019). B Cell-Based Cancer Immunotherapy. Transfus Med Hemother.

[45] Olingy CE, Dinh HQ, Hedrick CC (2019). Monocyte heterogeneity and functions in cancer. J Leukoc Biol.

[46] Wylie B, Macri C, Mintern JD, Waithman J (2019). Dendritic Cells and Cancer: From Biology to Therapeutic Intervention. Cancers (Basel).

[47] Varricchi G, Galdiero MR, Loffredo S, Marone G, Iannone R, Marone G (2017). Are Mast Cells MASTers in Cancer?. Front Immunol.

[48] Ettinger DS, Wood DE, Aisner DL, Akerley W, Bauman JR, Bharat A (2022). Non-Small Cell Lung Cancer, Version 3.2022, NCCN Clinical Practice Guidelines in Oncology. J Natl Compr Canc Netw.

[49] Memon H, Patel BM (2019). Immune checkpoint inhibitors in non-small cell lung cancer: A bird's eye view. Life Sci.

[50] Hassin O, Oren M (2023). Drugging p53 in cancer: one protein, many targets. Nat Rev Drug Discov.

[51] Havel JJ, Chowell D, Chan TA (2019). The evolving landscape of biomarkers for checkpoint inhibitor immunotherapy. Nat Rev Cancer.

[52] Liu J, Chen Z, Li Y, Zhao W, Wu J, Zhang Z (2021). PD-1/PD-L1 Checkpoint Inhibitors in Tumor Immunotherapy. Front Pharmacol.

[53] Latchman Y, Wood CR, Chernova T, Chaudhary D, Borde M, Chernova I (2001). PD-L2 is a second ligand for PD-1 and inhibits T cell activation. Nat Immunol.

[54] Van Coillie S, Wiernicki B, Xu J (2020). Molecular and Cellular Functions of CTLA-4. Adv Exp Med Biol.

[55] Ruffo E, Wu RC, Bruno TC, Workman CJ, Vignali DAA (2019). Lymphocyte-activation gene 3 (LAG3): The next immune checkpoint receptor. Semin Immunol.

[56] Zhao L, Cheng S, Fan L, Zhang B, Xu S (2021). TIM-3: An update on immunotherapy. Int Immunopharmacol.

[57] Harjunpaa H, Guillerey C (2020). TIGIT as an emerging immune checkpoint. Clin Exp Immunol.

[58] Woo SR, Turnis ME, Goldberg MV, Bankoti J, Selby M, Nirschl CJ (2012). Immune inhibitory molecules LAG-3 and PD-1 synergistically regulate T-cell function to promote tumoral immune escape. Cancer Res.

[59] Koyama S, Akbay EA, Li YY, Herter-Sprie GS, Buczkowski KA, Richards WG (2016). Adaptive resistance to therapeutic PD-1 blockade is associated with upregulation of alternative immune checkpoints. Nat Commun.

